# The current situation in compliance with hand hygiene in dental surgeries in the Czech Republic

**DOI:** 10.1186/2047-2994-4-S1-P151

**Published:** 2015-06-16

**Authors:** E SedlatáJurásková, I Matoušková

**Affiliations:** 1 Department of Orthodontics, Faculty of Medicine and Dentistry, Palacký University Olomouc, Olomouc, Czech Republic; 2 Department of Preventive Medicine, Faculty of Medicine and Dentistry, Palacký University Olomouc, Olomouc, Czech Republic

## Introduction

Implementation and compliance with hand hygiene in health care is different and depends on the type of separation. Hand Care is very important for the prevention of transmission of infectious agents between patients and dentist’s protection against infection. Their microbial contamination occurs when bioaerosol contacts hands and during a treatment of the patient. The literature indicates that the degree of “compliance” in compliance with hand hygiene in a dental surgery is between 16-81%, with an average of 40%.

## Objectives

The authors report in their work the current knowledge about the compliance policy guidance “Hand hygiene in health care” among dentists in the Czech Republic.

## Methods

Epidemiological investigation was carried out by a single anonymous questionnaire in a total number of 54 respondents.

## Results

Most were surprised to find that two dentists in private practice do not wash their hands before and after use non-sterile gloves.

We consider as highly positive finding that dentists in private dental surgeries in 88.89% used to disinfect hands disinfectants based on alcohol. In Faculty of Medicine and Dentistry, Palacký Univerzity Olomouc and Faculty Hospital Olomouc, which is under the supervision of the constitutional Hygienist, disinfectants for hands are in 100% alcohol-based.

## Conclusion

Hand hygiene has long been considered one of the most important infection control measures to prevent health care-associated infections. It is necessary to educate health care workers in dentistry about hand care.

## Disclosure of interest

None declared.

